# Interferon‐γ‐induced HLA Class II expression on endothelial cells is decreased by inhibition of mTOR and HMG‐CoA reductase

**DOI:** 10.1002/2211-5463.12854

**Published:** 2020-04-15

**Authors:** Akihiro Maenaka, Iwasaki Kenta, Akinobu Ota, Yuko Miwa, Wataru Ohashi, Kosei Horimi, Yutaka Matsuoka, Masafumi Ohnishi, Kazuharu Uchida, Takaaki Kobayashi

**Affiliations:** ^1^ Department of Pharmacy Aichi Medical University School of Medicine Nagakute Japan; ^2^ Department of Renal Transplant Surgery Aichi Medical University School of Medicine Nagakute Japan; ^3^ Department of Kidney Disease and Transplant Immunology Aichi Medical University School of Medicine Nagakute Japan; ^4^ Department of Biochemistry Aichi Medical University School of Medicine Nagakute Japan; ^5^ Division of Biostatistics Clinical Research Center Aichi Medical University School of Medicine Nagakute Japan

**Keywords:** CIITA, endothelial cell, everolimus, fluvastatin, HLA class II

## Abstract

In organ transplantation, donor‐specific HLA antibody (DSA) is considered a major cause of graft rejection. Because DSA targets primarily donor‐specific human leukocyte antigen (HLA) expressed on graft endothelial cells, the prevention of its expression is a possible strategy for avoiding or salvaging DSA‐mediated graft rejection. We examined the effect of various clinically used drugs on HLA class II expression on endothelial cells. Interferon‐γ (IFN‐γ)‐induced HLA class II DR (HLA‐DR) was downregulated by everolimus (EVR, 49.1% ± 0.8%; *P* < 0.01) and fluvastatin (FLU, 33.8% ± 0.6%; *P* < 0.01). Moreover, the combination of EVR and FLU showed a greater suppressive effect on HLA‐DR expression. In contrast, cyclosporine, tacrolimus, mycophenolic acid, and prednisolone did not exhibit any significant suppressive effect. FLU, but not EVR, suppressed mRNA of HLA‐DR. Imaging analysis revealed that HLA‐DR expressed in cytosol or on the cell surface was repressed by EVR (cytosol: 58.6% ± 4.9%, *P* < 0.01; cell surface: 80.9% ± 4.0%, *P* < 0.01) and FLU (cytosol: 19.0% ± 3.4%, *P* < 0.01; cell surface: 48.3% ± 4.8%, *P* < 0.01). These data indicated that FLU and EVR suppressed IFN‐γ‐induced HLA‐DR expression at the transcriptional and post‐translational level, respectively, suggesting a potential approach for alleviating DSA‐related issues in organ transplantation.

## Graphical abstract

Donor human leukocyte antigen (HLA)s on graft endothelial cells are targeted by donor‐specific HLA antibody (DSA), which is considered to be a major cause of antibody‐mediated rejection in organ transplantation. In this manuscript, it is suggested that the mTOR and HMG‐CoA reductase inhibitors suppress IFN‐γ‐induced HLA‐DR in endothelial cells. This finding may become the basis of a new approach for alleviating DSA‐related problems in organ transplantation.

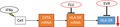

**Abbreviations**
CNI: calcineurin inhibitor
CSA: cyclosporine A
EVR: everolimus
FLU: fluvastatin
HLA: human leukocyte antigen
MPA: mycophenolic acid
PRD: prednisolone
TAC: tacrolimus

A factor that currently inhibits long‐term engraftment is chronic antibody‐mediated rejection (ABMR) by donor‐specific human leukocyte antigen (HLA) antibody (DSA), especially *de novo* DSA. Although mismatches of HLA class I and II have long been considered to increase the risk of acute rejection and graft failure [1, 2], chronic ABMR has been caused mainly by *de novo* DSA against HLA class II [3, 4]. Among HLA class II DSA, we have previously shown that *de novo* DSA against HLA class II DR (HLA‐DR) from the donor, rather than HLA class II DQ (HLA‐DQ), was significantly associated with chronic ABMR in renal transplantation [5, 6]. In general, HLA class II is expressed in B cells, macrophages, and dendritic cells, all of which have antigen‐presenting ability. In nonimmune cells such as endothelial cells, the expression level is limited during quiescent periods but is upregulated in an activated state such as inflammation [7].

During the maintenance period after transplantation, immunosuppressive therapy consists of multidrug combinations, and among them, calcineurin inhibitors (CNI), such as cyclosporine A (CSA) and tacrolimus (TAC), mycophenolate mofetil (MMF), and EVR are subjected to routine therapeutic drug monitoring, and the dosage is adjusted according to blood concentration [8, 9]. However, such multidrug immunosuppressive regimens frequently cause hyperlipidemia as an adverse effect of CNI (CSA and TAC) or EVR [10, 11]. A 3‐hydroxy‐3‐methyl‐glutaryl‐coenzyme A (HMG‐CoA) reductase inhibitor, so‐called ‘statin’, has received the most attention and has been widely used to treat solid organ transplant recipients with CNI ‐based regimens [12]. Although recent multidrug combination therapy has drastically reduced the incidence of acute rejection after transplantation, improvement of long‐term graft survival, to which ABMR is one of major obstacles, remains stagnant [13].

Because chronic ABMR is caused by an antibody–antigen reaction, we hypothesized that reduction of antigen expression could contribute to the treatment as well as antibody removal. In fact, the elimination of galactose‐α‐1,3‐galactose antigens, which could be the major target antigens in xenografts, raised hopes for pig‐to‐human xenotransplantation as a more realistic option with progress in genetic engineering technologies [14]. The use of tissue, cells, and organs from pigs avoids both hyperacute and humoral xenograft rejection without the need for complement inhibition or antibody absorption. Recently, researchers have attempted to eliminate or reduce swine leukocyte antigen (SLA) class I and class II because of the possibility of cross‐reactivity of DSA in patients sensitized against HLA and SLA [15].

In this study, we sought to determine which of the drugs clinically used after transplantation had an inhibitory effect on IFN‐γ‐induced HLA‐DR expression. EVR and FLU repressed interferon‐γ (IFN‐γ)‐induced HLA class II in EA.hy926 cells and human umbilical vein endothelial cells (HUVECs). Other immunosuppressive drugs did not show any repressive function on it. The combination of EVR and FLU showed additive effect on HLA class II expression.

## Materials and methods

### Cell culture and materials

EA.hy926 cells, the human endothelial‐like immortalized cell line derived from the fusion of HUVEC with the lung carcinoma cell line A549, were established as previously described [16]. EA.hy926 cells were maintained in Dulbecco’s modified Eagle’s medium, supplemented with 10% FBS (HyClone, Logan, UT, USA). HUVECs were obtained from the Lonza Corporation (Walkersville, MD, USA) and cultured in endothelial cell growth medium 2 (Lonza Corporation). IFN‐γ was purchased from R&D Systems (https://www.rndsystems.com/).

### Flow cytometry

EA.hy926 cells were incubated for 30 min at 4 °C with FITC‐labeled anti‐HLA‐DR antibody or PE‐labeled anti‐HLA‐DQ antibody (BioLegend, San Diego, CA, USA). Stained cells were then washed twice with phosphate‐buffer saline and analyzed with the FACSCanto II system (Becton Dickinson, San Jose, CA, USA). The expression rate of HLA‐DR suppressed by EVR or FLU was calculated according to the following formula ‘[(M.F.I of EVR or FLU)/ (M.F.I of IFN‐γ only)] × 100’ (%).

### Cell apoptosis analysis

EA.hy926 cells were treated with drugs or cultured without FBS for 72 h. Then, cells were incubated for 5 min at room temperature with FITC‐labeled annexin V and PI (BD, Franklin Lakes, NJ, USA). Annexin V‐positive apoptotic cell was measured by FACS.

### Quantitative real‐time PCR

Total RNA was extracted from cells using the QIAzol Lysis Reagent and the miRNeasy Mini Kit (Qiagen, Hilden, Germany). Quantitative real‐time PCR was carried out with an iCycler system (Bio‐Rad, Hercules, CA, USA). Total RNA was reverse‐transcribed with 1‐μm oligo (dT) primers and high‐capacity reverse transcriptase (Takara, Tokyo, Japan), according to the manufacturer's instructions (Step 1: 25 °C 10 min; Step 2: 37 °C, 120 min: Step 3: 85 °C, 5 min). Complementary DNA of class II transactivator (CIITA) and HLA‐DR was amplified [Step 1: 95 °C, 10 s; Step 2 (40 cycles): 95 °C, 15 s and 60 °C, 30 s; Step 3: 95 °C, 60 s; Step 4: 58 °C, 30 s; and Step 5: 95 °C, 30 s] and normalized to the level of glyceraldehyde 3‐phosphate dehydrogenase (GAPDH). Expression level of CIITA or HLA‐DR mRNA was calculated by delta‐delta Ct method and described as a relative value of control (IFN‐γ treatment). Quantitative real‐time PCR was performed in the Mx3000P quantitative PCR system (Stratagene, La Jolla, CA, USA) using SYBR Premix Ex Taq (Takara Bio Inc., Shiga, Japan). Primers were obtained from Rikaken CO., LTD. (Nagoya, Japan). Primer sequences were indicated as follows. GAPDH: Forward, ATCCCTGAGCTGAACGGGAA; Reverse, TGTCATACCAGGAAATGAGCTTGA. CIITA: Forward, AGACACCATCAACTGCGACC; Reverse, GCGATATTGGCATAAGCCTCC. HLA‐DR: Forward, GTTTACGACTGCAGGGTGGA; Reverse, CCATCACCTCCATGTGCCTT.

### Western blotting

Western blots were performed with whole‐cell lysates. Antibodies against tubulin and HLA‐DR (Abcam PLC, Cambridge, UK) were used with a working dilution in Can Get Signal solution I (Toyobo, Tokyo, Japan), and secondary antibodies (anti‐rabbit IgG horseradish peroxidase from Cell Signaling, Danvers, MA, USA) were used at 1 : 2000 dilutions in Can Get Signal solution II (Toyobo). Signals were observed via ECL Western blotting Detection Reagents (GE Healthcare UK Ltd., Buckinghamshire, UK). The western blot images were captured using the Amersham Imager 600 (GE Healthcare UK Ltd.). Quantification (%) was calculated according to the following formula ‘[(band intensity of HLA‐DR)/ (band intensity of Tubulin bands)] × 100’ (%) by using imagej software (NIH, Bethesda, MD, USA).

### Imaging cytometry

EA.hy926 cells were fixed with formaldehyde and permeability buffer (Thermo Fisher Scientific, Indianapolis, IN, USA) as a pretreatment. The cells were washed with phosphate‐buffer saline and incubated with FITC‐labeled anti‐HLA class II antibody (LN3) (BioLegend) and CellMask (Thermo Fisher Scientific, Chino, CA, USA) for 30 min at room temperature. After washing, to stain cell nuclei, DAPI solution (Dojindo Laboratories, Kumamoto, Japan) was added to the cells for 10 min at room temperature. Stained cells were then washed three times with phosphate‐buffer saline and analyzed with the IN Cell Analyzer 6000 (GE Healthcare UK Ltd.). To analyze the intensity of HLA‐DR in the cell membrane and cytosol, configuration of the membrane thickness was set after the erosion process from the cell surface based on the specified cytoplasm area stained by CellMask.

### Statistical analysis

The results are represented as mean ± standard errors of the mean. Statistical analysis was performed by the Dunnett methods for multiple comparison test with the use of the stat view‐j 5.0 (SAS Institute Inc., Cary, NC, USA). A *P* value of less than 0.05 or less than 0.01 was considered statistically significant.

## Results

### EVR and FLU suppressed IFN‐γ‐induced expression of HLA‐DR

**Fig. 1:**
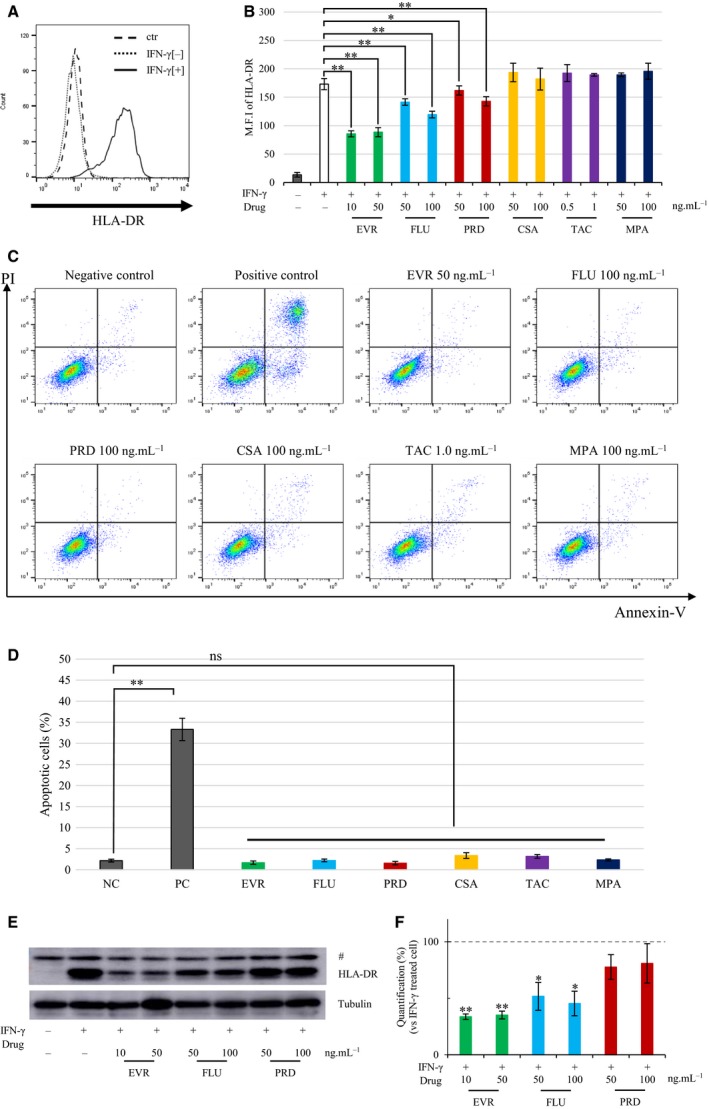
The suppressive effect of immunosuppressive drugs on IFN‐γ‐induced HLA‐DR. EA.hy926 cells were preincubated with the drugs for 30 min, followed by 100 ng·mL^−1^ of IFN‐γ stimulation for 72 h. Cells were then subjected to flow cytometry. (A) Representative flow cytometry was conducted with isotype immunoglobulin G–fluorescein isothiocyanate (IgG‐FITC) as the control condition (ctr; dashed line), and anti‐HLA‐DR‐FITC against endothelial cells not treated with IFN‐γ (IFN‐γ[−]; dotted line) and those treated with IFN‐γ (IFN‐γ[+]; solid line). (B) EA.hy926 cells were preincubated with EVR (10 or 50 ng·mL^−1^), FLU (50 or 100 ng·mL^−1^), PRD (50 or 100 ng·mL^−1^), CSA (50 or 100 ng·mL^−1^), TAC (0.5 or 1.0 ng·mL^−1^), or MPA (50 or 100 ng·mL^−1^) for 30 min, followed by 100 ng·mL^−1^ of IFN‐γ stimulation for 72 h. Cells were harvested, and HLA‐DR expression was measured by flow cytometry. The white bar represents control value of mean fluorescence intensity (M.F.I.) (*n* = 4). (C, D) EA.hy926 cells were treated with 50 ng·mL^−1^ EVR, 100 ng·mL^−1^ FLU, 100 ng·mL^−1^ PRD, 100 ng·mL^−1^ CSA, 1.0 ng·mL^−1^ TAC, or 100 ng·mL^−1^ MPA for 72 h. Untreated cells were indicated as the negative control, and cells cultured with FBS‐free medium were indicated as the apoptotic cells (positive control). Apoptotic cells were analyzed by annexin V/PI staining assay. Representative flow cytometry figure (C) and the percentage of apoptotic cells (annexin V‐positive cells) (D) were shown (*n* = 3). (E, F) EA.hy926 cells were preincubated with the drugs for 30 min, followed by 100 ng·mL^−1^ IFN‐γ stimulation for 72 h. Cells were harvested and subjected to western blot analysis with anti‐HLA‐DR antibody. Representative western blotting figure (E) and quantification (F) (*n* = 3) are shown, and # indicates nonspecific bands. Data represent mean ± SEM. Dunnett’s test *P < 0.05, **P < 0.01.

**Fig. 2:**
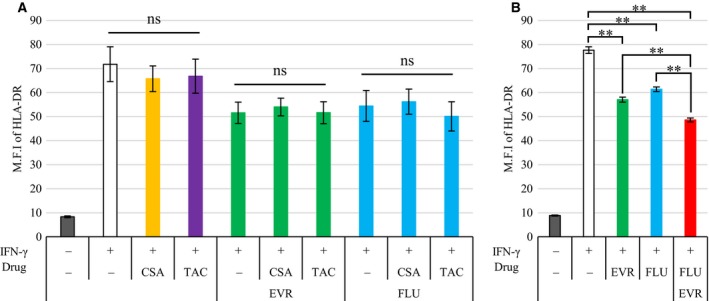
The suppressive effect by EVR and FLU on IFN‐γ‐induced HLA‐DR in the presence of CNI. (A) EA.hy926 cells were preincubated with 50 ng·mL^−1^ of EVR or 100 ng·mL^−1^ of FLU together with 100 ng·mL^−1^ CSA or 1.0 ng·mL^−1^ TAC for 30 min, followed by 100 ng·mL^−1^ of IFN‐γ stimulation for 72 h. Cells were harvested, and HLA‐DR expression was measured by flow cytometry. The white bar represents control value of M.F.I. (*n* = 3). (B) EA.hy926 cells were preincubated with 50 ng·mL^−1^ of EVR and/or 100 ng·mL^−1^ of FLU for 30 min, followed by 100 ng·mL^−1^ of IFN‐γ stimulation for 72 h. Cells were harvested, and HLA‐DR expression was measured by flow cytometry. The white bar represents control value of M.F.I. (*n* = 3). Data represent mean ± SEM. Dunnett’s test **P < 0.01.

Because quiescent endothelial cell lines do not express HLA‐DR, we induced its expression in endothelial cells by stimulating them with IFN‐γ (Fig. 1A). Cells were preincubated with the drugs for 30 min, followed by IFN‐γ stimulation for 72 h. We used EVR, FLU, prednisolone (PRD), cyclosporine (CSA), TAC, and mycophenolic acid (MPA). Flow cytometry analysis revealed that IFN‐γ‐induced HLA‐DR expression was downregulated by EVR and FLU (Fig. 1B). PRD had moderately suppressive effects, but CSA, TAC, and MPA did not (Fig. 1B). The rates of downregulation by EVR (50 ng·mL^−1^) and FLU (100 ng·mL^−1^) were 49.1% ± 0.8% and 33.8% ± 0.6%, respectively, which was statistically different as compared to IFN‐γ‐treated cells. Apoptosis analysis revealed that all of the drugs did not lead to apoptosis within the range of concentrations used in this study (Fig. 1C,D). Western blot analysis revealed clear downregulation of EVR and FLU (Fig. 1E,F). Considering the results of flow cytometry and western blots, EVR and FLU demonstrated preventive effects on IFN‐γ‐induced HLA‐DR expression in endothelial cells. Since multidrug usage of immunosuppressive drugs is commonly conducted in organ transplantation, combination experiments were conducted to evaluate whether EVR and FLU could suppress IFN‐γ‐induced HLA‐DR expression in endothelial cells in the presence of CNI. Cells were preincubated with EVR and/or FLU together with CNI for 30 min, followed by IFN‐γ stimulation for 72 h. Flow cytometry analysis revealed that IFN‐γ‐induced HLA‐DR expression was downregulated by EVR and FLU even in the presence of CNI (Fig. 2A). Further analysis revealed that the combination of EVR and FLU additively repressed IFN‐γ‐induced HLA‐DR expression (Fig. 2B). Taken together, EVR and FLU, but not other immunosuppressive drugs, have the preventive effect on IFN‐γ‐induced HLA‐DR expression on endothelial cells.

### EVR post‐translationally suppressed IFN‐γ‐induced expression of HLA‐DR

**Fig. 3:**
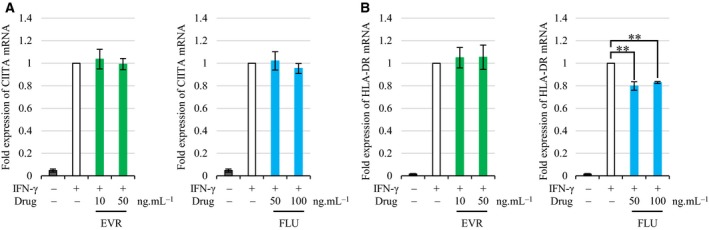
The effect of EVR and FLU on transcriptional regulation of CIITA and HLA‐DR mRNA. (A, B) EA.hy926 cells were preincubated with EVR (10 or 50 ng·mL^−1^) or FLU (50 or 100 ng·mL^−1^) for 30 min, followed by 100 ng·mL^−1^ of IFN‐γ stimulation for 24 h. Total RNA was purified and subjected to quantitative real‐time polymerase chain reaction to measure the mRNA expression of CIITA (A) and HLA‐DR alpha (B). The fold expression of CIITA or HLA‐DR is indicated in comparison with endothelial cells treated with IFN‐γ as control (1.0; white bar) (*n* = 4). Data represent mean ± SEM. Dunnett’s test **P < 0.01.

**Fig. 4:**
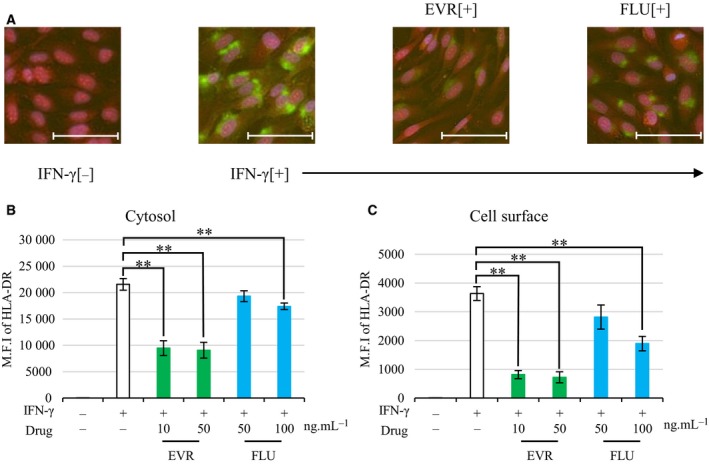
The suppressive effect of EVR and FLU on IFN‐γ‐induced HLA‐DR expression in cytosol and on cell surface. EA.hy926 cells were preincubated with EVR (10 or 50 ng·mL^−1^) or FLU (50 or 100 ng·mL^−1^) for 30 min, followed by 100 ng·mL^−1^ of IFN‐γ stimulation for 72 h. Cells were subjected to imaging cytometry with FITC‐labeled anti‐HLA‐DR antibody, CytoTell dye, and DAPI. (A) Representative imaging figures are shown in the presence of EVR and FLU. The length of the scale bars is 30 μm. (B, C) The effect of EVR, 10 or 50 ng·mL^−1^, or FLU, 50 or 100 ng·mL^−1^, on IFN‐γ‐induced HLA‐DR distribution in cytosol (B) and on cell surface (C). Data represent mean ± SEM (*n* = 4). Dunnett’s test **P < 0.01.

To reveal the mechanism of the suppressive effect of EVR and FLU on IFN‐γ‐induced HLA‐DR, we examined mRNA expression of CIITA and HLA‐DR. Cells were preincubated with the drugs for 30 min, followed by IFN‐γ stimulation for 24 h. Total RNA was purified from samples 24 h after IFN‐γ stimulation, and quantitative real‐time polymerase chain reaction was conducted to measure mRNA of CIITA and HLA‐DR. Neither drug affected IFN‐γ‐induced CIITA transcription (Fig. 3A). FLU, but not EVR, suppressed HLA‐DR expression by mRNA (Fig. 3B). To investigate HLA‐DR expression further, we used imaging cytometry to measure its location in the cells. HLA‐DR was upregulated on cell surface after IFN‐γ stimulation. Figure 4A shows that IFN‐γ‐induced HLA‐DR was expressed both in cytosol and on cell surface. EVR suppressed IFN‐γ‐induced HLA‐DR expression both in cytosol (suppression rate of 58.6% ± 4.9%; *P* < 0.01) and on cell surface (suppression rate of 80.9% ± 4.0%; *P* < 0.01) (Fig. 4B,C). FLU suppressed IFN‐γ‐induced HLA‐DR expression on cell surface (suppression rate of 48.3% ± 4.8%; *P* < 0.01) and also suppressed in cytosol (suppression rate of 19.0% ± 3.4%; *P* < 0.01) when 100 ng·mL^−1^ of FLU was used (Fig. 4B,C). These results indicated that EVR‐mediated downregulation of HLA‐DR expression might be caused by post‐translational regulation.

### EVR suppressed IFN‐γ‐induced HLA‐DQ expression

**Fig. 5:**
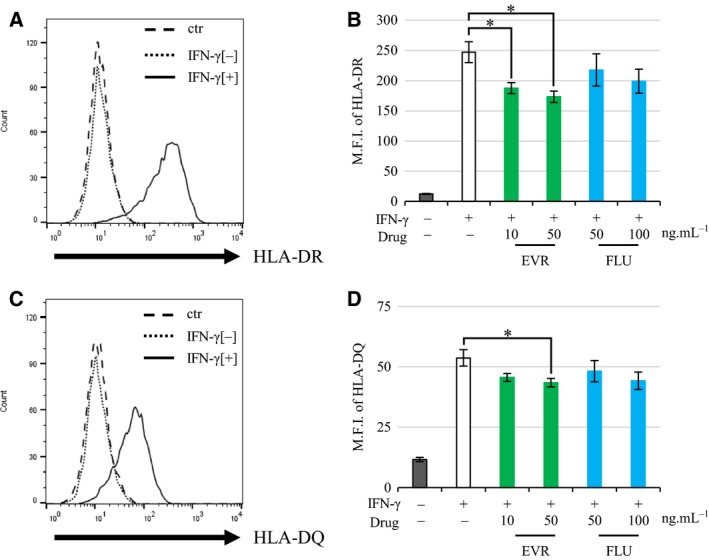
The effect of EVR and FLU on IFN‐γ‐induced HLA‐DQ expression. (A, C) HUVECs were treated with 10 ng·mL^−1^ IFN‐γ for 72 h. Cells were subjected to flow cytometry. Representative flow cytometry results are shown with isotype IgG‐FITC/PE as the control (ctr; dashed line), and anti‐HLA‐DR FITC (A)/anti‐HLA‐DQ‐PE (C) against endothelial cells either not treated with IFN‐γ (IFN‐γ[−]; dotted line) or treated with IFN‐γ (IFN‐γ[+]; solid line). The representative results of flow cytometry were shown. (B, D) HUVECs were preincubated with EVR (10 or 50 ng·mL^−1^) or FLU (50 or 100 ng·mL^−1^) for 30 min, followed by 100 ng·mL^−1^ of IFN‐γ stimulation for 72 h. Cells were subjected to flow cytometry with anti‐HLA‐DR (B) and HLA‐DQ (D). Data represent mean ± SEM (*n* = 3). Dunnett’s test *P < 0.05.

Next, we conducted an experiment to clarify whether EVR and FLU also suppressed HLA‐DQ. Because HLA‐DQ was not expressed in EA.hy926 endothelial cells, we used HUVECs. Since HUVECs also do not express HLA class II in a quiescent state, it was stimulated with IFN‐γ. Cells were preincubated with the drugs for 30 min, followed by IFN‐γ stimulation for 72 h. HLA‐DR and HLA‐DQ were measured by flow cytometry. Not only HLA‐DR (Fig. 5A) but also HLA‐DQ (Fig. 5C) expression was induced by IFN‐γ. While both HLA‐DR expression and HLA‐DQ expression were suppressed by EVR (DR; 29.6% ± 3.0%; *P* < 0.05, DQ; 18.7% ± 2.7%; *P* < 0.05), FLU did not statistically repress both HLA‐DR and HLA‐DQ expression (Fig. 5B,D). Taken together, these results indicated that EVR would have the potential to downregulate HLA class II expression on endothelial cells in inflammatory conditions.

## Discussion

It has been reported that DSA can cause irreversible ABMR and lead to graft dysfunction. However, antirejection therapy such as plasmapheresis, intravenous immunoglobulin, rituximab, and bortezomib has successfully prevented acute ABMR for patients with preformed or *de novo* DSA [17]. After occurrence of ABMR, however, the treatment could reverse the rejection during the induction phase but not sufficiently at the maintenance phase, which suggests that no medical treatment is effective in preventing chronic ABMR [6, 18, 19]. The reduction of HLA‐DR and HLA‐DQ expression may be an alternative strategy for avoiding chronic ABMR after *de novo* DSA production, because the majority of DSA are directed to HLA‐DQ and/or DR [5, 6]. We previously reported that anti‐blood group A ligation decreased IFN‐γ‐induced HLA‐DR expression on endothelial cells through inactivation of the mTOR pathway [20]. In this study, results indicate that EVR and FLU clinically used after transplantation have a potential to downregulate HLA class II expression (Figs 1, 2, 3, 4), which may give effective strategy against preformed DSA and *de novo* DSA. Previous reports have indicated that a switch from CNI to EVR (withdraw CNI) showed more frequent DSA appearance and increased risk of ABMR than the standard regimen (with CNI) [21, 22, 23]. Results of a recent comparative study between ‘EVR with reduced‐exposure CNI (EVR + rCNI)’ and ‘mycophenolate with standard‐exposure CNI’ have demonstrated lowered incidence of *de novo* DSA and similar cellular rejection rate in the EVR + rCNI arm among on‐treatment patients [24]. Although EVR might have the potential to reduce DSA‐induced humoral rejection, we need to carefully assess the efficacy of EVR considering combined use of CNI.

Professional antigen‐presenting cells (APCs) effectively internalize antigen by phagocytosis or endocytosis and then display a peptide fragment from the antigen bound to a major histocompatibility complex (MHC) class II molecule on cell surface, where CD4 T cells recognize and interact with the antigen–MHC class II molecule [25]. Recently, the antigen presentation by HLA class II on non‐APCs has attracted attention, especially with regard to autoimmune diseases and cancer. MHC class II molecules bind with intact misfolded proteins without peptide processing, are transported to the cell surface, and are then recognized by antigen‐specific B cells, which results in the antibody production against self‐antigens [26, 27]. The evaluation of tumor‐infiltrating CD4 T cells has revealed that locally expressed MHC class II tumor‐associated antigen showed gain of function to eliminate tumor cells in the microenvironment [28]. In this study, EVR decreased expression of IFN‐γ‐induced HLA‐DR both in cytosol and on cell surface (Fig. 4), which indicates that EVR might have advantage for autoimmune disease.

CSA, TAC, PRD, and MMF are commonly used as immunosuppressive drugs, and we investigated whether these drugs could also regulate IFN‐γ‐induced HLA‐DR expression. PRD prevented IFN‐γ‐induced HLA‐DR expression to a lesser extent than EVR and FLU (Fig. 1). CSA was reported to reduce the expression of adhesion molecules and MHC class II in the kidneys of rats [29]. However, the concentration used in that study was extremely high, far exceeding the clinical dosage. Further investigation would be needed, but we do not currently expect CSA to have a preventive effect on HLA‐DR expression at clinical blood levels. EVR did not prevent CIITA or HLA‐DR mRNA transcription (Fig. 3), but it did inhibit IFN‐γ‐induced HLA‐DR protein expression both in cytosol and on cell surface post‐translationally (Fig. 4). FLU is a statin, a HMG‐CoA reductase inhibitor, used in the treatment of dyslipidemia [30]. Because dyslipidemia is a common complication after renal transplantation [31], statins are frequently used in transplant recipients [32]. We obtained the suppressive effect of FLU on HLA‐DR expression in the same way as EVR (Fig. 1), which is consistent with the previous report that statins suppress IFN‐γ‐induced MHC class II [33, 34]. However, those reports also suggested that statins have a suppressive effect on CIITA mRNA expression, whereas our study showed that the HLA‐DR expression was suppressed at the level of HLA‐DR mRNA but not CIITA mRNA (Fig. 3). EVR troughs were maintained in the range between 3 and 8 ng·mL^−1^ in the first post‐transplant year [35]. In comparison, twice‐daily administration of 0.75 or 1.5 mg of EVR gave the maximum concentration (*C*_max_) at a level higher than that by 20 ng·mL^−1^ of EVR [36]. In the case of FLU, *C*_max_ reached an average of 443 ng·mL^−1^ after administration of 40 mg of FLU [37]. Therefore, we assumed that the concentrations of EVR and FLU used in cell culture would be within the clinically effective range. In addition, the combination of EVR and FLU showed additive effect on HLA‐DR expression on cell surface. In any event, it is possible that the statins could be regulators of HLA‐DR mRNA transcription.

Everolimus may also have a suppressive effect against HLA‐DQ expression (Fig. 5) in HUVECs. However, its effectiveness was weaker than that observed in EA.hy926 cells. The reason might be ascribed to the difference in responsiveness against IFN‐γ between EA.hy926 cells (100 ng·mL^−1^ of IFN‐γ) and HUVECs (10 ng·mL^−1^ of IFN‐γ). The limitations of this study are that we did not consider the difference in reactivity of IFN‐γ and the drugs used in this study between EA.hy926 and HUVECs.

In this study, we have found that the induction of HLA‐DR expression in endothelial cells can be suppressed by two drugs commonly used after renal transplantation: EVR and FLU. Furthermore, the downregulation mechanisms of the two agents differed: FLU suppressed transcription, and EVR influenced post‐translational modification. Further investigation is needed to verify their suppressive effect on HLA class II in actual renal graft *in vivo*. With this in mind, our findings would provide useful information regarding the selection of immunosuppressive drugs to overcome ABMR caused by DSA.

## Conflict of interest

The authors declare no conflict of interest.

## Author contributions

AM, KI, and TK designed the study, generated the hypothesis, analyzed the data, and wrote the manuscript. AM, KI, YM, WO, and AO performed the experiments, interpreted the results, and wrote the manuscript. KH, YM, MO, and KU reviewed the manuscript.
